# Different conformational responses of the β_2_-adrenergic receptor-Gs complex upon binding of the partial agonist salbutamol or the full agonist isoprenaline

**DOI:** 10.1093/nsr/nwaa284

**Published:** 2020-11-24

**Authors:** Fan Yang, Shenglong Ling, Yingxin Zhou, Yanan Zhang, Pei Lv, Sanling Liu, Wei Fang, Wenjing Sun, Liaoyuan A Hu, Longhua Zhang, Pan Shi, Changlin Tian

**Affiliations:** Hefei National Laboratory of Physical Sciences at Microscale and School of Life Sciences, University of Science and Technology of China, Hefei 230026, China; Hefei National Laboratory of Physical Sciences at Microscale and School of Life Sciences, University of Science and Technology of China, Hefei 230026, China; Hefei National Laboratory of Physical Sciences at Microscale and School of Life Sciences, University of Science and Technology of China, Hefei 230026, China; Hefei National Laboratory of Physical Sciences at Microscale and School of Life Sciences, University of Science and Technology of China, Hefei 230026, China; Hefei National Laboratory of Physical Sciences at Microscale and School of Life Sciences, University of Science and Technology of China, Hefei 230026, China; Hefei National Laboratory of Physical Sciences at Microscale and School of Life Sciences, University of Science and Technology of China, Hefei 230026, China; Hefei National Laboratory of Physical Sciences at Microscale and School of Life Sciences, University of Science and Technology of China, Hefei 230026, China; Hefei National Laboratory of Physical Sciences at Microscale and School of Life Sciences, University of Science and Technology of China, Hefei 230026, China; Amgen Asia R&D Center, Amgen Research, Shanghai 201210, China; Hefei National Laboratory of Physical Sciences at Microscale and School of Life Sciences, University of Science and Technology of China, Hefei 230026, China; Hefei National Laboratory of Physical Sciences at Microscale and School of Life Sciences, University of Science and Technology of China, Hefei 230026, China; Hefei National Laboratory of Physical Sciences at Microscale and School of Life Sciences, University of Science and Technology of China, Hefei 230026, China; High Magnetic Field Laboratory, Chinese Academy of Sciences, Hefei 230030, China

**Keywords:** cryo-EM structure, G protein-coupled receptor (GPCR), partial and full agonists, conformational change, desensitization

## Abstract

G protein-coupled receptors (GPCRs) are responsible for most cytoplasmic signaling in response to extracellular ligands with different efficacy profiles. Various spectroscopic techniques have identified that agonists exhibiting varying efficacies can selectively stabilize a specific conformation of the receptor. However, the structural basis for activation of the GPCR-G protein complex by ligands with different efficacies is incompletely understood. To better understand the structural basis underlying the mechanisms by which ligands with varying efficacies differentially regulate the conformations of receptors and G proteins, we determined the structures of β_2_AR-Gα_s_$\beta $γ bound with partial agonist salbutamol or bound with full agonist isoprenaline using single-particle cryo-electron microscopy at resolutions of 3.26 Å and 3.80 Å, respectively. Structural comparisons between the β_2_AR-Gs-salbutamol and β_2_AR-Gs-isoprenaline complexes demonstrated that the decreased binding affinity and efficacy of salbutamol compared with those of isoprenaline might be attributed to weakened hydrogen bonding interactions, attenuated hydrophobic interactions in the orthosteric binding pocket and different conformational changes in the rotamer toggle switch in TM6. Moreover, the observed stronger interactions between the intracellular loop 2 or 3 (ICL2 or ICL3) of β_2_AR and Gα_s_ with binding of salbutamol versus isoprenaline might decrease phosphorylation in the salbutamol-activated β_2_AR-Gs complex. From the observed structural differences between these complexes of β_2_AR, a mechanism of β_2_AR activation by partial and full agonists is proposed to provide structural insights into β_2_AR desensitization.

## INTRODUCTION

G protein-coupled receptors (GPCRs) regulate a wide variety of physiological functions in response to extracellular stimuli. The varying efficacies of agonists binding to the receptor mediate distinct interaction networks in the orthosteric site, and preferentially stabilize different active conformational states of GPCRs [1–4]. The different conformations of receptors promote binding and activation of different downstream signaling effectors, such as G proteins and β-arrestins, leading to a wide range of intracellular signaling profiles, referred to as efficacy profiles [5–7]. Biophysical studies have indicated that ligands with different efficacy profiles stabilize distinct receptor conformations [8–10], but these conformations and the mechanism by which ligands induce them have not been fully understood.

Notably, GPCR conformational changes caused by binding of agonists with varying efficacies not only reflect the efficacy of the agonist but also induce GPCR desensitization [11], that is, decreased receptor responses to continuous agonist stimulation [12,13]. Numerous studies have shown that the process of GPCR desensitization involves multiple steps, including protein kinase A (PKA)-mediated receptor phosphorylation of intracellular loop 3 (ICL3), G protein receptor kinase (GRK)-mediated receptor phosphorylation in the intracellular loops and the C-terminal tail (C-tail), β-arrestin binding to the receptor, and receptor endocytosis or recycling [13–16]. Among these mechanisms, phosphorylation of ICL3 was observed to induce uncoupling of the receptor from the Gs complex [12], eventually leading to desensitization. Functional and biophysical studies demonstrated that partial agonist binding caused less GPCR desensitization than full agonist binding [14,17,18]. Notably, GPCR desensitization plays crucial roles in modulating receptor activation, which is also essential for analyzing the pharmacokinetics of drugs targeting GPCR. However, the structural basis of GPCR desensitization induced by partial or full agonists is still elusive and needs to be addressed.

**Figure 1.**
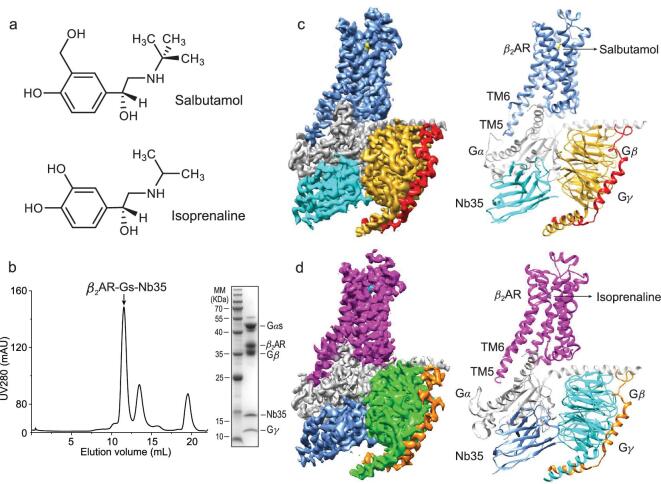
Cryo-EM structure of salbutamol or isoprenaline-bound β_2_AR-Gs complex. (a) Chemical structures of salbutamol and isoprenaline. (b) Size exclusive chromatography and SDS-PAGE profile of the purified salbutamol-β_2_AR-Gs complex. (c) Cryo-EM density map and ribbon diagram representation of the cryo-EM structure of salbutamol (yellow), β_2_AR (blue), Gαs Ras-like (grey), Gβ (gold), Gγ (red), and Nb35 (cyan). (d) Cryo-EM density map and ribbon diagram representation of the cryo-EM structure of isoprenaline (cyan), β_2_AR (magenta), Gαs Ras-like (grey), Gβ (green), Gγ (orange), and Nb35 (blue).

The β_2_ adrenergic receptor (β_2_AR) is a prototypical family A GPCR. Salbutamol (albuterol) is a rapid-onset, short-acting, selective partial agonist of β_2_AR over β_1_AR, which is located in the heart; thus, its cardiac toxicity is minimized [19,20] (Fig. 1a). More interestingly, salbutamol is a functionally selective β_2_AR partial agonist that is biased toward Gs over arrestin [21], which may prevent arrestin-dependent proinflammatory effects. These pharmacological properties of salbutamol have contributed to its successful use in treating asthma and chronic obstructive pulmonary disease (COPD). Isoprenaline is a full agonist that has shown higher intrinsic efficacy and a stronger bias toward β-arrestin recruitment than salbutamol (Fig. 1a). Interestingly, continuous agonist stimulation induces GPCR desensitization [13]. A strong correlation was found between the coupling efficiencies of the agonists and their ability to induce desensitization; for example, compared with the full agonist isoprenaline, the partial agonist salbutamol caused greater reductions in the initial rates of phosphorylation and β-arrestin recruitment and significantly reduced desensitization [14,22]. In addition, recent studies have revealed that G protein and β-arrestin compete for overlapping binding sites in the GPCR transmembrane core [11]. To illustrate the structural foundation of β_2_AR activation and desensitization upon binding of partial or full agonists, we sought to determine the three-dimensional structures of the β_2_AR-Gα_s_$\beta $γ complex bound with a partial agonist salbutamol or a full agonist isoprenaline via single-particle cryo-EM.

## RESULTS

### Structures of the salbutamol- and isoprenaline-bound **β**_2_AR-Gs complexes

**Figure 2.**
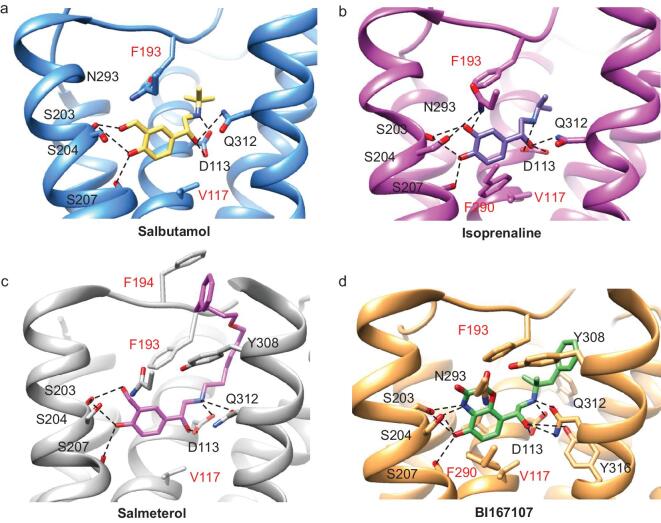
Comparison of agonist-binding modes of partial agonists and full agonists. Side views of the orthosteric binding pockets in the salbutamol-bound (yellow) (a), isoprenaline-bound (purple) β_2_AR-Gs complex (b), salmeterol-bound (magenta) β_2_AR-Nb71 complex (c) and BI167107-bound (green) β_2_AR-Gs complex (d). Residues within 4 Å of all ligands are shown as sticks and the hydrogen bond interactions are represented by dotted lines. Red and blue sticks represent oxygen and nitrogen, respectively. The hydrophobic amino acid residues are shown in red.

The cryo-EM structures of the partial agonist salbutamol- and the full agonist isoprenaline-bound β_2_AR-Gs complexes were determined at 3.26 Å and 3.80 Å resolution, respectively (Fig. 1 and Figs S1–S7). The partial agonist (salbutamol) or full agonist (isoprenaline) was clearly identified in the orthosteric binding site of β_2_AR. The global folds of salbutamol–bound β_2_AR-Gs and isoprenaline–bound β_2_AR-Gs were similar (Fig. 2a and b). Comparison of these structures with that of the inactive-state β_2_AR, which was bound with the antagonist carazolol, revealed outward movement of TM6, suggesting that both salbutamol- and isoprenaline-bound β_2_AR are in an active state (Fig. 4a) [23,24].

### Notable differences in interactions within the orthosteric binding site of partial agonists and full agonists

Different binding interfaces of the partial or full agonists in the orthosteric binding site of β_2_AR might indicate different activation mechanisms [25]. The β_2_AR-Gs complex structure (3SN6), activated by the ultrahigh affinity agonist BI167107, represents the fully active state of β_2_AR [23]. To better understand different agonist-induced conformational changes, we compared the structures of the ligand binding pockets of β_2_AR bound to the full agonists BI167107 or isoprenaline with those of β_2_AR bound to the partial agonists salbutamol or salmeterol. All four agonists bound in similar orthosteric sites. Moreover, all the head groups of the agonists can form hydrogen bonds with S203^5.42^ and S207^5.46^ [26], and all of the β-hydroxyl groups on the agonists form hydrogen bonds with D113^3.32^ (Fig. 2). Notably, three major differences were observed in the agonist binding pocket when the full agonists were bound compared to when the partial agonists were bound. First, structural differences in S204^5.43^ and N293^6.55^ were observed (Fig. 2). Specifically, N293^6.55^ forms a hydrogen bond with S204^5.43^ in isoprenaline-bound β_2_AR, and this hydrogen bond plays an important role in stabilizing ligand binding (Fig. 2b). However, this hydrogen bond interaction is not observed in the salbutamol-bound β_2_AR-Gs complex, apparently because of the rotameric conformational change in S204^5.43^ in salbutamol-bound β_2_AR (Fig. 2a). In addition, a hydrogen bond is observed between N293^6.55^ and the *meta*-hydroxyl of isoprenaline, but is not present between N293^6.55^ and salbutamol. The cAMP accumulation functional assay combined with alanine mutagenesis revealed that mutation of residues S204^5.43^ or S293^6.55^ substantially reduced isoprenaline potency and signaling but had little effect on salbutamol function (Fig. S8, Table S3). The observed result is consistent with previous functional studies of these residues, which verified that mutation of S204^5.43^ and N293^6.55^ induced decreases in Gs activation and β-arrestin recruitment [27]. These indicated that the attenuated hydrogen bond interactions in the salbutamol-bound β_2_AR structure might be responsible for the reduced affinity and desensitizing effect of the partial agonist salbutamol compared with the full agonist isoprenaline (Fig. 2a and b). Secondly, a significant difference between the partial and full agonists is that attenuated hydrophobic interactions are formed only between the aromatic ring of salbutamol/salmeterol and residues of β_2_AR through V117^3.36^ and F193^ECL2^ (Fig. 2a and c), while hydrophobic interactions are formed between isoprenaline/BI167107 and residues of β_2_AR through V114^3.33^ and V117^3.36^ in TM3, F193 in ECL2 and F290^6.52^ in TM6 (Fig. 2b and d). Therefore, the decreased interaction of salbutamol versus isoprenaline with residues in the binding pocket of β_2_AR might cause the weakened binding affinity and reduced activation, resulting from salbutamol binding to β_2_AR compared with isoprenaline binding to β_2_AR. The structural comparisons of the ligand binding pockets of β_2_AR for salbutamol and salmeterol demonstrated highly similar interactions, verifying our observed interactions between salbutamol and residues in the orthosteric binding site of β_2_AR (Fig. 2a and c and Fig. S9). Thirdly, K305^7.32^ in the salbutamol-bound β_2_AR forms a hydrogen bond with D192^ECL2^. However, K305^7.32^ in the BI167107-bound β_2_AR-Gs complex trades its salt bridge with D192^ECL2^ for an interaction with the backbone carbonyl of F193^ECL2^, stabilizing its movement toward Y308^7.35^ to form a lid over the orthosteric binding site (Fig. S10) [28]. The lid obstructs ligand association and dissociation (Fig. 2d). The distance between Y308^7.35^ and F193^ECL2^ in the salbutamol-bound β_2_AR-Gs complex is longer than that in the BI167107-bound state, further suggesting the low affinity and partial activation effect of salbutamol (Fig. S10). Although both isoprenaline and BI167107 are full agonists, BI167107 interacts more strongly than the isoprenaline with β_2_AR (Fig. 2b and d). In isoprenaline-bound β_2_AR, the side chain of K305^7.32^ moves but still interacts with F192^ECL2^, and it did not cause Y308^7.35^ to move toward the ligand (Fig. S10). The cAMP accumulation assay revealed that alanine substitution of residues K305 and F193 in the isoprenaline binding pocket decreased the potency of isoprenaline (Fig. S10b, Table S3), which confirmed that these residues played important roles in the isoprenaline-mediated cAMP signaling pathway.

**Figure 3.**
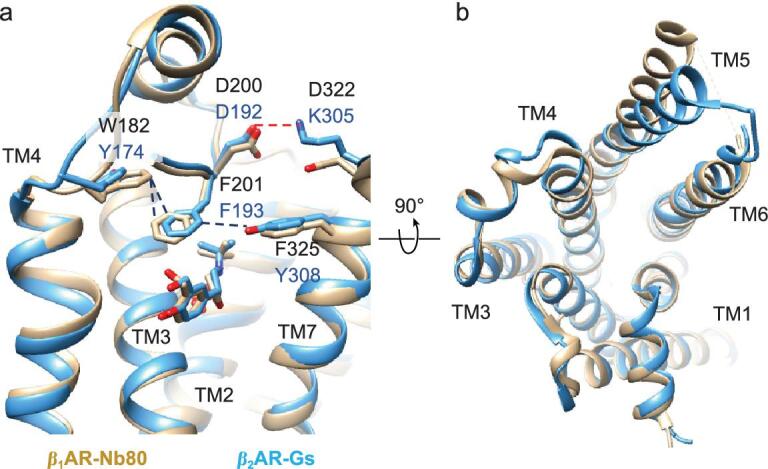
Comparison of the salbutamol-bound β_1_AR and β_2_AR. Side views (a) and intracellular views (b) of the salbutamol-bound β_1_AR-Nb80 (gold) and β_2_AR-Gs (blue) complex. Interactions between amino acids are indicated by dotted lines.

Most strikingly, salbutamol exhibits a selectivity of approximately 20-fold for β_2_AR over β_1_AR [19]. We compared the structure of the salbutamol-bound β_2_AR-Gs complex with that of the salbutamol-bound β_1_AR-Nb80 complex (PDB : 6H7M) [29]. In the salbutamol-bound β_1_AR-Nb80 complex, W182^ECL2^ interacts with F201^ECL2^ and causes F201^ECL2^ to move away from F325^7.35^ (Fig. 3). However, in the salbutamol-bound β_2_AR-Gs complex, F193^ECL2^ is within the van der Waals distance of Y308^7.35^ on the opposite side of the entrance to the orthosteric binding pocket, which had a major effect on decreasing the rates of ligand association and dissociation. Moreover, an electrostatic interaction is formed between D192^ECL2^ and K305^7.32^ in β_2_AR, which was not observed in β_1_AR (Fig. 3a and b). Because of these interactions, dissociation of salbutamol from β_2_AR is more difficult.

### Conformational variations in the intracellular side of salbutamol- and isoprenaline-bound β_2_AR

**Figure 4.**
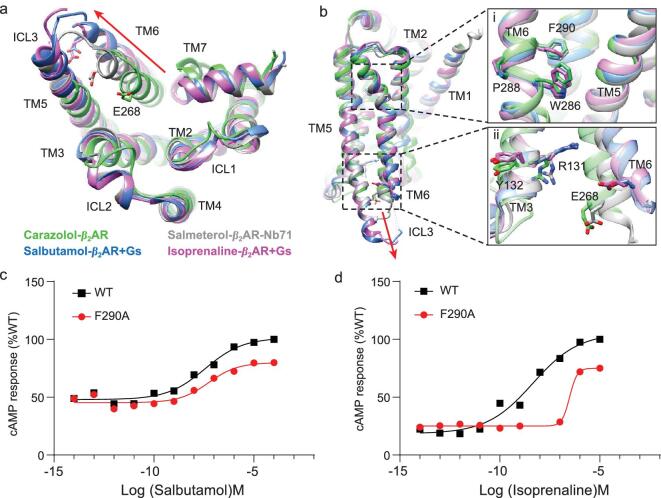
The notable conformational changes of the transmembrane helical bundle. (a) Comparison of the cytoplasmic view of transmembrane helical bundle conformation in the carazolol-bound β_2_AR (green), the salmeterol-bound β_2_AR-Nb71 (gray), the salbutamol (blue) and the isoprenaline- (magenta) bound β_2_AR-Gs complex. The red arrow shows the position of TM6 relative to the helical bundle (the Cα of Glu268^6.30^ as a reference). (b) Comparison of the side view of transmembrane domain conformation in the carazolol-bound β_2_AR (green), the salmeterol-bound β_2_AR-Nb71 (gray), the salbutamol (blue) and the isoprenaline- (magenta) bound β_2_AR-Gs complex. Enlarged view of the conserved core of the receptors (right), the rotamer toggle switch (b-i) and ionic lock (b-ii) are presented, respectively. (c and d) cAMP accumulation assay of F290A in the rotamer toggle switch in the salbutamol-bound and isoprenaline-bound β_2_AR.

The intracellular side of the receptor in the salbutamol- and isoprenaline-bound β_2_AR-Gs structures exhibit marked conformational differences relative to the structures of the inverse agonist carazolol-bound β_2_AR complex and the highly potent partial agonist salmeterol-bound β_2_AR complex. As shown in Fig. 4, relative to the inactive state carazolol-β_2_AR structure, the displacement of the cytoplasmic end of TM6 in the salbutamol-bound β_2_AR-Gs structure (14.1 Å) is larger than in the salmeterol-bound β_2_AR-Nb71 structure (8 Å), but slightly smaller than that in the isoprenaline-bound β_2_AR structure (14.6 Å) when measured at the Cα carbon of E268^6.30^ (Fig. 4a). Two molecular switches have been reported

 

to be associated with receptor activation and to be responsible for the movement of TM6. The first one is the rotamer toggle switch, referred to as the rotamer configurations of Cys285^6.47^, Trp286^6.48^ and Phe290^6.52^, which are coupled and modulate the bend angle of TM6 around the highly conserved proline kink at Pro288^6.50^, leading to movement of the cytoplasmic end of TM6 upon activation [30,31]. The other molecular switch that plays a decisive role in TM6 movement is the ionic lock, which is defined on the cytoplasmic end of the receptor [32]. In the inactive state, R131^3.50^ on TM3 forms a salt bridge with D130^3.49^ and E268^6.30^ on TM6, which is disrupted upon agonist binding, causing TM6 to be released and to move away from TM3 (Fig. 4b) [23]. The side chains of Phe290^6.52^ in the isoprenaline-β_2_AR structure undergo significant rotation relative to their positions in the carazolol-β_2_AR and salbutamol-β_2_AR structure to form the bending angle of Pro288^6.50^, and the side chains of R131^3.50^ and E268^6.30^ in isoprenaline-bound β_2_AR move up and disrupt the ionic lock (Fig. S11). Thus, both the rotamer toggle switch and the ionic lock switch exist in isoprenaline-β_2_AR, which is a hallmark of GPCR activation, leading to the largest movement of TM6 (14.6 Å) and contributing to its high efficacy. However, in salbutamol-bound β_2_AR, salbutamol does not trigger the rotamer toggle switch in TM6 but only disrupts the ionic lock between TM3 and TM6, thus leading to a smaller movement of TM6 (14.1 Å) and contributing to its lower efficacy. The local density maps of the rotamer toggle switch in the salbutamol or isoprenaline-bound β_2_AR-Gs complex are shown in Fig. S11 a and b. The cAMP accumulation assay revealed that mutation of the residue F290 to Ala reduced isoprenaline activated signaling but had little effect on salbutamol function (Fig. 4c and d, Table S3). In the salmeterol-bound β_2_AR-Nb71 structure (PDB : 6MXT), the rotamer toggle is not triggered, and the ionic lock still exists, resulting in the smallest movement of TM6 (8 Å), which indicates the importance of Gs protein binding for β_2_AR activation (Fig. 4a and b).

### Intracellular loops mediate different G protein-activated conformations upon partial agonist and full agonist binding

**Figure 5.**
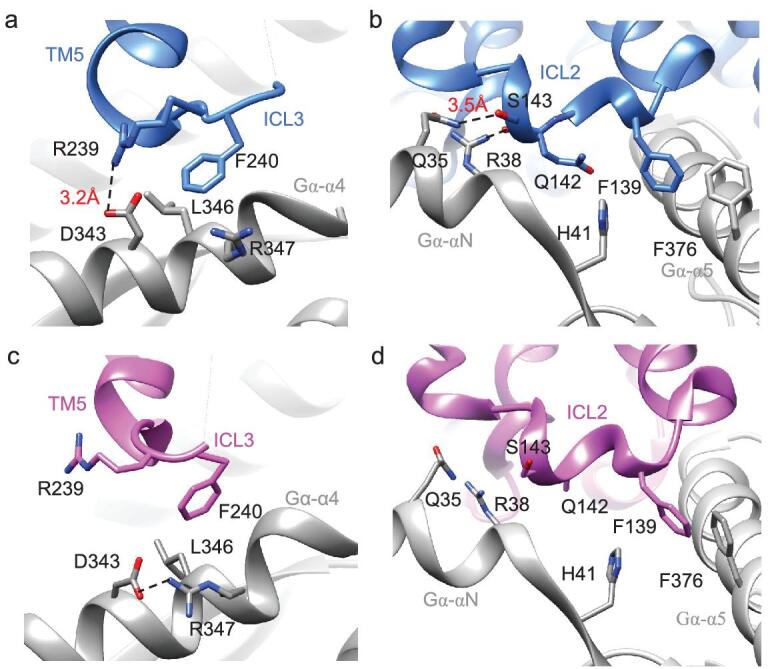
Structural comparison of intracellular loops conformation between salbutamol- and isoprenaline-bound β_2_AR-Gs. The interaction interface between ICL3 (a), ICL2 (b) of β_2_AR and Gs protein in the salbutamol-bound β_2_AR-Gs complex. The interaction interface between ICL3 (c), ICL2 (d) of β_2_AR and Gs protein in the isoprenaline-bound β_2_AR-Gs complex.

Structural comparisons between the salbutamol-β_2_AR-Gs and isoprenaline-β_2_AR-Gs complexes also demonstrated that binding of partial or full agonists to β_2_AR led to different conformations in the intracellular region and different interaction interfaces with the Gα_s_βγ complex. Our cryo-EM density map allowed us to define the interaction between ICL3 and Gαs (Fig. 5). As shown in Fig. 5a, F240 in ICL3 in salbutamol-bound β_2_AR forms a hydrophobic interaction with L346 in the Gα-α4 helix. The side chain of R239 in ICL3 is 3.2 Å away from D343 in the α4 helix of the G protein and can form electrostatic interactions. In the structure of the isoprenaline-β_2_AR-Gs complex, D343 flips, which keeps D343 away from R239 of ICL3 (Fig. 5c). When the partial agonist salbutamol binds to β_2_AR, β_2_AR-ICL3 interacts more strongly with the G protein than when the full agonist isoprenaline binds. Moreover, β_2_AR-ICL2 becomes more tightly bound to the Gs protein. In addition to the hydrophobic pockets formed by F139^ICL2^ and F376 in α5 helix that have been observed in the isoprenaline-β_2_AR-Gs complex, residue Q35 in the αN helix of Gαs forms a polar interaction with S143^ICL2^ in the receptor in the salbutamol-β_2_AR-Gs complex. Residue R38 in αN helix also forms a polar interaction with the main chain carbonyl oxygen of Q142 in ICL2, which was not observed in the isoprenaline-β_2_AR-Gs complex (Fig. 5c and d and Fig. S12). These structural characteristics indicate that the interaction between the β_2_AR-Gs interface in the partial agonist salbutamol-bound β_2_AR-Gs complex is enhanced compared with that in the full agonist isoprenaline-bound β_2_AR-Gs complex. The stronger interaction between the receptor and G protein in the salbutamol-β_2_AR-Gs complex might make it difficult to expose the phosphorylation site in the loop, which probably contributes to the decreased desensitizing effect of salbutamol compared with isoprenaline.

## DISCUSSION

Functional and biophysical approaches, such as nuclear magnetic resonance spectroscopy and single-molecule fluorescence technology, have demonstrated that partial and full agonists induce distinct active conformations of GPCRs [10,33,34]. Our work provides the structural basis for the different conformational changes in the GPCR-G protein complex evoked by partial or full agonists. We propose two significant determinants that affect the difference in the agonist efficacy between the partial agonist salbutamol and the full agonist isoprenaline: the weakening of agonist interactions with the orthosteric binding site for salbutamol, and the less successful induction of conformational changes involving the rotamer toggle switch and the ionic lock switch on the intracellular side of salbutamol-bound β_2_AR. Although in the absence of G protein, full agonists and partial agonists will cause different degrees of conformational changes at the C-terminus of TM6 of the receptor. However, when the receptor binds to the G protein, it can make the TM6 of the receptor reach a fully activated state, no matter whether combined with full agonists or partial agonists.

**Figure 6.**
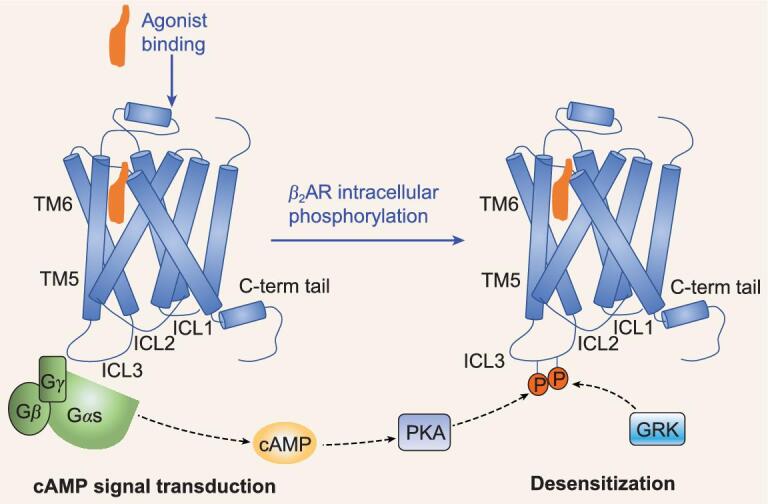
Diagrammatic model representation of receptor desensitization regulated by agonist.

GPCR desensitization was previously proposed through phosphorylation of ICL3 and the C-tail on the GPCR, uncoupling of G proteins, binding of β-arrestin to the receptor, and GPCR internalization or endocytosis [13–16] (Fig. 6). Accumulated previous studies have illustrated that ICL3 could play an important role in G protein coupling and receptor phosphorylation [13,35,36]. Therefore, we hypothesized a model for the mechanism by which partial agonists induced less desensitization based on the structures of the salbutamol- and isoprenaline-bound β_2_AR-Gs complexes. Specifically, notable differences, including attenuated hydrogen bonds and hydrophobic interactions, were observed in the ligand binding pockets following treatment with the partial agonist salbutamol compared to the full agonist isoprenaline. A recent study reported that residues in the allosteric ligand binding pocket regulate GPCR interactions with β-arrestin [37]. Herein, it is observed that β_2_AR-ICL3 could interact more tightly with the G protein during binding of the partial agonist salbutamol, than binding of the full agonist isoprenaline. Therefore, phosphorylation of ICL3 could be more difficult in the salbutamol-β_2_AR-Gs complex, which might contribute to the decreased desensitization, triggered by salbutamol binding versus isoprenaline binding.

A previous study indicated that agonists promote GPCR phosphorylation not only at ICL3 and the C-tail but also at the first and second intracellular loops [38]. In addition, the formoterol-bound β_1_AR-arrestin complex demonstrates that both the ICL1 and ICL2 loops in β_1_AR engage in the interaction with arrestin [39]. Relative to its position in the β_1_AR-arrestin/formoterol complex, the amino acid in ICL2 that is involved in the binding of both the G protein and arrestin is in a different conformational state. This observation further illustrates the role of ICL2 in receptor activation and desensitization (Fig. S13). However, as the C-tail of β_2_AR was truncated at residue K348 in this expression construct, we could not observe the configuration around consensus substrate sites phosphorylated by GRKs. Herein, combinations of the structural comparisons and function assays of β_2_AR-Gs complex bound with isoprenaline or salbutamol indicate that the increased interaction interface between the β_2_AR and Gs protein in the salbutamol-β_2_AR-Gs structure attenuates agonist-dependent receptor phosphorylation, which could lead to the reduced β_2_AR desensitizing effect of the partial agonist salbutamol. Therefore, structural and cAMP assays in this work suggest a framework for different extents of β_2_AR desensitization upon binding of partial or full agonist. Further structural and functional studies are required to elucidate detailed mechanisms of arrestin-mediated desensitization upon partial or full agonist binding.

## CONCLUSION

We report the cryo-EM structures of the β_2_AR-Gs complex bound to the partial agonist salbutamol or the full agonist isoprenaline. Comparison of salbutamol-bound β_2_AR with isoprenaline-bound β_2_AR revealed notable differences in the ligand binding pockets. First, the interaction between S204^5.43^ and N293^6.55^ is eliminated and a hydrogen bond is formed between salbutamol and N293^6.55^ in the salbutamol-β_2_AR structure relative to the isoprenaline-β_2_AR structure. Second, hydrophobic interactions between the salbutamol aromatic ring and β_2_AR are attenuated compared with those between isoprenaline and β_2_AR. We speculate that these collective structural differences in ligand binding pockets might account for the decreased affinity of the partial agonist salbutamol compared with the full agonist isoprenaline. Moreover, unlike isoprenaline, salbutamol does not trigger the rotamer toggle switch in TM6 but only disrupts the ionic lock between TM3 and TM6, contributing to its lower efficacy. In addition, the stronger interactions between the β_2_AR-Gs protein binding interface in the partial agonist salbutamol-bound β_2_AR-Gs complex might decrease phosphorylation in the salbutamol-activated β_2_AR-Gs complex, contributing to weaker β-arrestin binding and lower desensitization. Thus, this work provides structural insights into the differences in GPCR activation between the partial agonist salbutamol and the full agonist isoprenaline and extends knowledge of agonist-induced desensitization, which is important for drug development and disease treatment.

## METHODS

### Expression and purification of human **β**_2_AR

The human β_2_AR truncated at the C-terminal from residue 348 was optimized as described previously [40,41]. The construct with FLAG tag at N-terminal and 10 × His at C-terminal was synthesized by GenScript and then cloned into pFastBac1 vector and was expressed in *Spodoptera frugiperda* Sf9 insect cells using the baculovirus method. The mutation E122W was introduced to improve thermostability of the receptor. The receptor was extracted from insect cell membranes with 1% n-dodecyl-β-D-maltopyranoside (DDM) and purified by TALON Metal Affinity Resin (Clontech). The eluted protein was concentrated and further purified by size-exclusion chromatography on a Superdex 200 10/300 GL column (GE Healthcare) (Supplementary data).

### Expression, purification of Nb35, G**α**s, G**βγ** and Gs complex reconstitution

Nanobody35 (Nb35) [32] was cloned into pET22b vector. The human Gαs was cloned into pET28a vector. They were expressed in *E.* *coli* (BL_21_(Gold)). The bovine Gβ_1_-C68S and N-terminal 6 × His tagged Gγ_2_ were cloned into pFastBac-Dual vector and expressed in Sf9 insect cells (Supplementary data).

### β_2_AR-Gs complex preparation

The β_2_AR and Gs complex proteins were mixed at a molar ratio 1 : 1.2. The mixed sample was incubated at room temperature for 1.5 h, then Apyrase was added. The mixture was incubated with 1% L-MNG to exchange the detergent, and Nb35 was added to further maintain the stability of the receptor-G protein complex. The protein complex was concentrated and further purified by size-exclusion chromatography on a Superdex 200 10/300 GL column (GE Healthcare).

### Cryo-EM sample preparation and data collection

An aliquot of 2.5 μL of the sample (0.5 mg/mL) was applied to plasma-treated (H_2_/O_2_, 10 s) grids (Quantifoil R1.2/1.3300-mesh Au Holey Carbon). The grids were blotted for 6 s at 100% humidity and 4°C.

Cryo-EM images were recorded on a Gatan K2 Summit direct electron detector in an FEI Titan Krios electron microscope at 300 kV. Serial-EM was used for automated data collection [42]. Movies were collected at a nominal magnification of 29 000 × in counting mode, corresponding to a pixel size of 1.014 Å.

### Image processing

For salbutamol-bound β_2_AR-Gs complex, a total of 7026 micrograph stacks were collected and subjected to motion correction using motioncor2 [43]. Contrast transfer function parameters were estimated with Gctf [44]. A 50 Å low-pass filtered 3D initial model de novo from the 2D average particles was generated using the stochastic gradient descent (SGD) algorithm in Relion-3.0 [45]. The 455 803 particles from the best-looking class were selected for 3D auto-refinement. By post-processing and particle polishing, the final resolution was improved to 3.26 Å. Map resolution was estimated with the gold-standard Fourier shell correction 0.143 criterion. Local resolution was estimated using Resmap [46].

For isoprenaline-bound β_2_AR-Gs complex, 702 049 particles from well-defined 2D averages were selected from 6217 micrographs. A selected subset of 231 827 particles was used to obtain the final map. The global resolution of this map was estimated to be 3.8 Å based on the gold-standard Fourier shell correlation (FSC).

Details on ‘Model building and refinement’ and ‘Functional analysis of cAMP assay’ are available in the Supplementary data.

Density maps and structure coordinates have been deposited in the Electron Microscopy Database and the Protein Data Bank with accession numbers 7DHI and 7DHR.

## Supplementary Material

nwaa284_Supplemental_File
