# Supplementary material for: Different conformational responses of the β2-adrenergic receptor-Gs complex upon binding of the partial agonist salbutamol or the full agonist isoprenaline
Source: Natl Sci Rev. 2020 Nov 24;8(9):nwaa284. doi: 10.1093/nsr/nwaa284 (PMC11261663; doi:10.1093/nsr/nwaa284)
Supplement: nwaa284_Supplemental_File [file nwaa284_Supplemental_File.docx]

**SUPPLEMENTARY INFORMATION FOR**

**Different Conformational Responses of the β_2_**-**Adrenergic Receptor-Gs Complex upon Binding of the Partial Agonist Salbutamol or the Full Agonist Isoprenaline**

Fan Yang^1^, Shenglong Ling^1^, Yingxin Zhou^1^, Yanan Zhang^1^, Pei Lv^1^, Sanling Liu^1^, Wei Fang^1^, Wenjing Sun^1^, Liaoyuan A. Hu^2^, Longhua Zhang^1^, Pan Shi^1^, Changlin Tian^1,3^

Correspondence: Shi Pan ([shipan@ustc.edu.cn](mailto:shipan@ustc.edu.cn)), or Changlin Tian ([cltian@ustc.edu.cn](mailto:cltian@ustc.edu.cn))

**This PDF file includes:**

Supplementary Methods

Supplementary Figures S1-S13

Supplementary Table S1-S3

**Supplementary Methods**

**Purification of human β2AR**

Cell pellets were resuspended and dounced in low salt buffer (10 mM HEPES, pH 7.5, 10 mM MgCl_2_, 20 mM KCl, protease inhibitors named cocktail (Sigma, S8820)). The suspension was ultracentrifuged at 180,000g for 45 min. Then the supernatant was discarded and membrane precipitate was resuspended and dounced in high salt buffer (10 mM HEPES, pH7.5, 10 mM MgCl_2_, 20 mM KCl, 1M NaCl, protease inhibitor cocktail). After centrifugation at 180,000g for 45 min, membrane pellets were resuspended in buffer (10 mM HEPES, pH7.5, 10 mM MgCl_2_, 20 mM KCl, 30% glycerol, protease inhibitor cocktail). To prepare ligand activated β_2_AR samples, 50 µM isoproterenol or 100 µM salbutamol were added and incubated at 4 °C for 1 hour. The insect membrane was solubilized with 1% n-Dodecyl-β-D-Maltopyranoside (DDM) at 4°C for 2 hours. The supernatant fraction isolated by centrifugation at 180,000 g for 45 min was collected and incubated with preprocessed TALON Metal Affinity Resin (Clontech) overnight at 4°C. The target protein was eluted in buffer consisting of 50 mM HEPES, PH 7.5, 0.01% DDM, 0.002% CHS, 150 mM NaCl, 300 mM IMD, 50 µM isoproterenol (or 100 µM Salbutamol). The eluted protein was concentrated and further purified by size-exclusion chromatography on Superdex 200 10/300 GL column (GE Healthcare).

**Purification of Nb35, Gαs, Gβγ and Gs complex reconstitution.**

Gαs and Gβγ share the same purification system. Cell pellets were resuspended and lysed by high pressure homogenizer. Then suspension was centrifugated at 14000 rpm for 30 min at 4°C. The preprocessed Ni-NTA affinity column (QIAGEN) was added into supernatants and incubated 40 min at 4°C. The resin was washed with washing buffer and eluted with buffer consisting of 50 mM HEPES, pH7.5, 200 mM NaCl, 250 mM imidazole, 5.6 mM β-ME. The eluted sample was then concentrated and further purified on a Superdex 200 10/300 GL column. Then, Gαs and Gβγ were mixed at molar ratio 1:1 and incubated at room temperature for 1 h to form Gαsβγ heterotrimeric complex. Gαsβγ complex was isolated on a Superdex 200 10/300 GL column (GE Healthcare).

Nanobody35 was cloned into pET22b vector, and expressed in *E.Coli* *(BL21(Gold)*) system. The expression and purify conditions of Nb35 refer to the Gαs. The eluted protein was concentrated and further purified by size-exclusion chromatography on Superdex 75 10/300 GL column (GE Healthcare).

**Model building and refinement**

The initial model for the salbutamol- and isoprenaline- bound β_2_AR-Gs complexes were derived from BI167107-bound β_2_AR-Gs complex (3SN6) followed by extensive remodeling using COOT (1). The N-terminal residues 1-29, residues 242-264 and C-terminal residues 341-413 of β_2_AR were not built due to the lack of corresponding densities. Structure refinements were carried out by PHENIX in real space with secondary structure and geometry restraints to prevent structure overfitting (2,3). Figures were prepared using UCSF Chimera or PyMOL(4).

**Functional analysis of cAMP assay**

The function data of β_2_AR was measured based on intracellular cAMP assay using cAMP-Gs dynamic kit (Csbio). HEK293T cells were cultured in DMEM medium supplemented with 10% (v/v) fetal bovine serum, 50 µg/mL penicillin and 50 μg/ml streptomycin using 37°C incubator supplied with 5% CO_2_. Cells were seeded onto 6-well cell culture plates before transfection. After overnight culture, the cells were transfected with β_2_AR-pcDNA3.1 plasmid (or β_2_AR mutation plasmid) using Lipofectamine 3000 transfection reagent (Invitrogen). About 12 h after transfection, the cells were collected, and suspended in DMEM containing 500 µM 3-isobutyl-1- methylxanthine (IBMX) (Sigma). Then, the cells were seeded onto 384-well plates at a density of 7500 cells per well. The transfected cells were incubated for 45 min with gradient concentrations of isoprenaline or salbutamol at incubator. Finally, the cells were incubated with cAMP-D2 and anti-cAMP Crypate for 1 h at room temperature and time-resolved FRET signals were measured at 620 nm and 650 nm using CLARIOstar plate reader (BMG LabTech, Germany).

**Supplementary Reference:**

1. Emsley, P., Lohkamp, B., Scott, W.G. & Cowtan, K. Features and development of Coot. *Acta Crystallogr.D.* 2010; **66**: 486-501.

2. Adams, P.D. et al. PHENIX: a comprehensive Python-based system for macromolecular structure solution. *Acta Crystallogr.D.* 2010; **66**: 213-21.

3. Afonine, P.V. et al. New tools for the analysis and validation of cryo-EM maps and atomic models. *Acta Crystallogr. D*. 2018; **74**: 814-840.

4. Pettersen, E.F. et al. UCSF Chimera--a visualization system for exploratory research and analysis. *J Comput Chem.* 2004; **25**: 1605-12.

**Supplementary Figures**


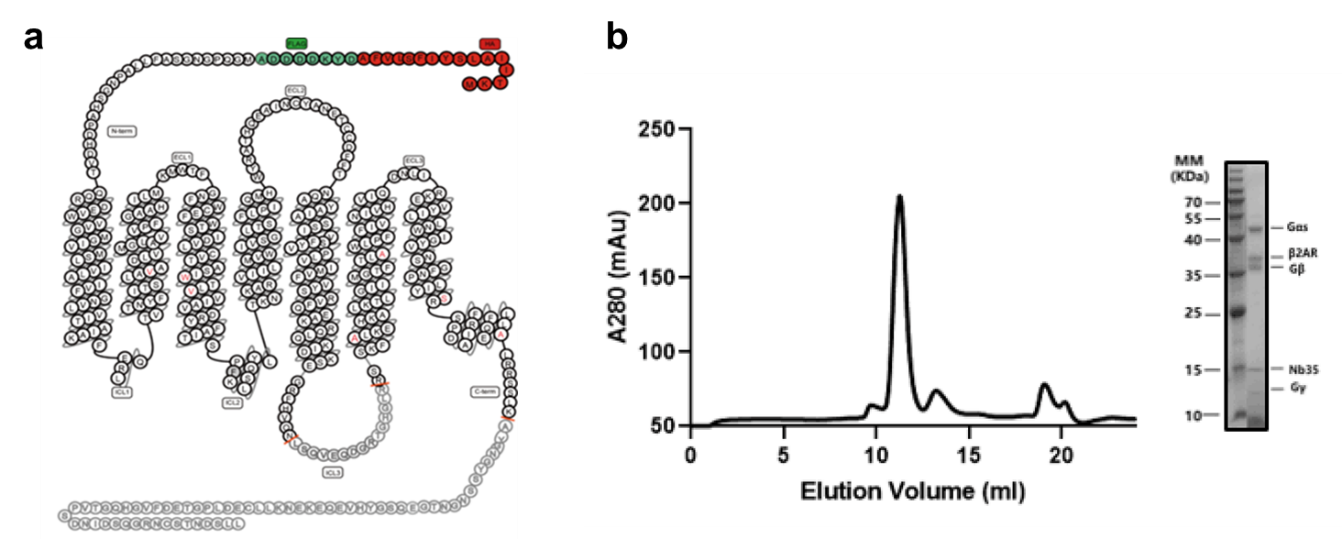


**Figure S1. Purification of isoprenaline bound β_2_AR-Gs complex.**

1. Schematic diagram of the β_2_AR used in the study. In our construct, HA signal peptide (red) and flag epitope (green) were linked at N-terminus. Seven residues colored in red were mutated for protein stabilization. The amino acids within ICL3 (△245-△259) and C terminus (△349-△413) were truncated to increase protein expression.
2. Size exclusive chromatography elution profile of the purified isoprenaline-β_2_AR-Gs complex and SDS-PAGE analysis of the corresponding complexe after elution.


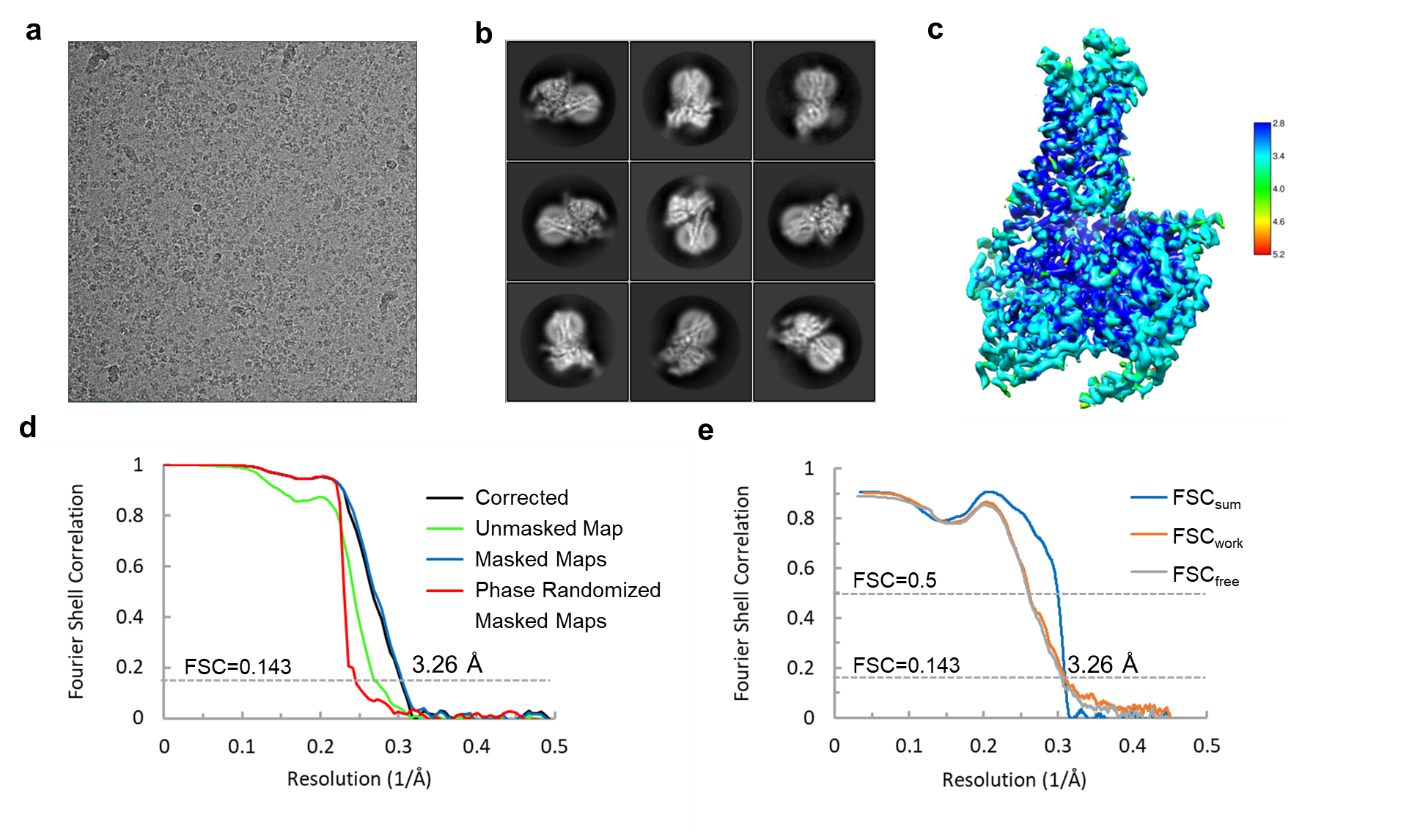


**Figure S2. Cryo-EM data analysis of human salbutamol-β_2_AR-Gs complex.**

1. A representative cryo-EM micrograph of β_2_AR-Gs-salbutamol complex after motion correction and dose weighting.
2. Representative 2D class averages of salbutamol-β_2_AR-Gs complex showing distinct secondary structure features from different views.
3. Cryo-EM density map colored by local resolution estimation using Resmap.
4. The gold-standard Fourier Shell Correlation curve of salbutamol-β_2_AR-Gs complex indicating the resolution at the FSC = 0.143 is 3.26Å.
5. FSC_work_/FSC_free_ validation curves of salbutamol-β_2_AR-Gs complex.


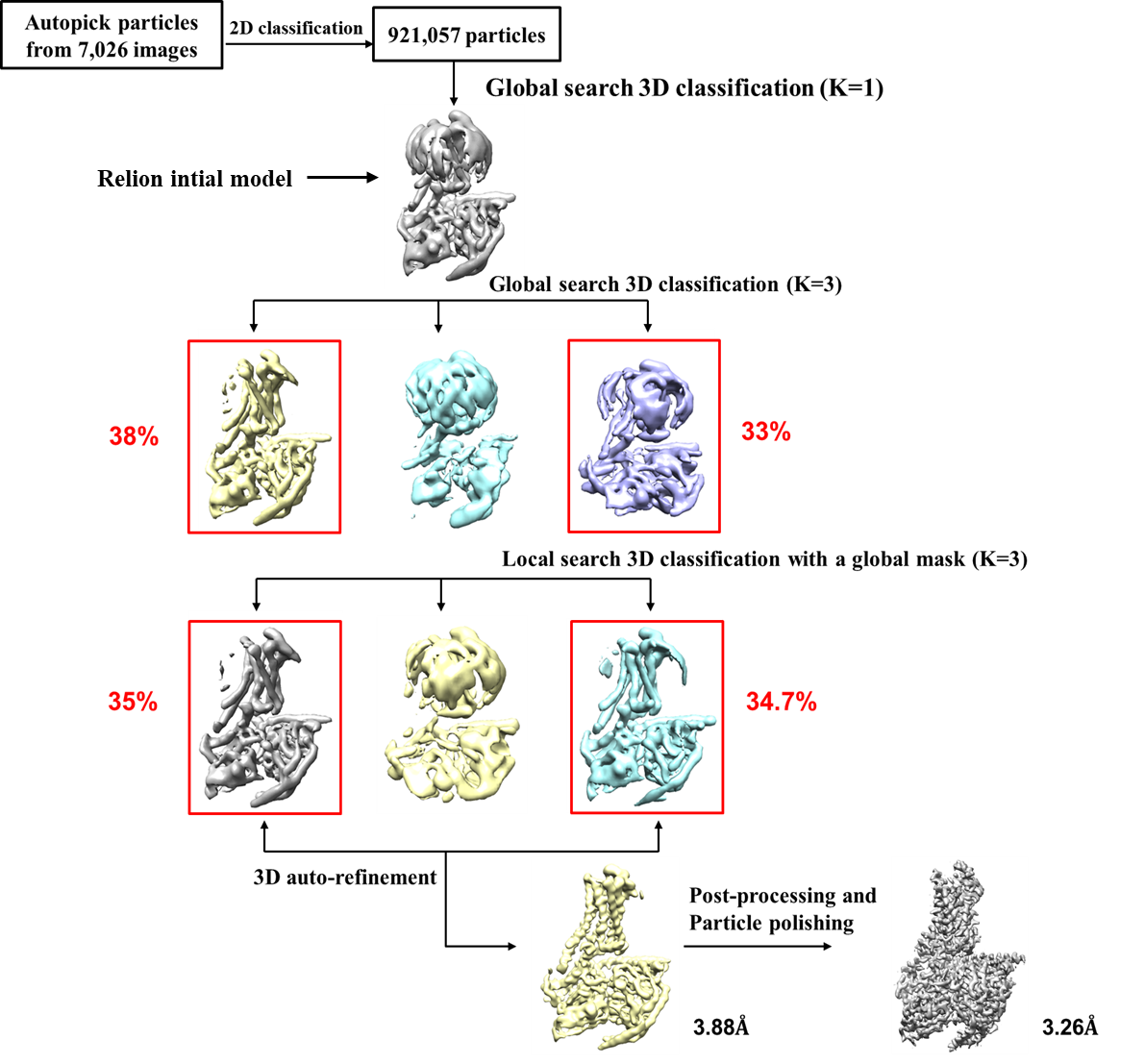


**Figure S3. A flowchart for the cryo-EM data processing and structure determination of the salbutamol-β_2_AR-Gs complex, including particle projection selection, classification and refinement.**

The final reconstructed density map has an average resolution of 3.26 Å. All the images in this figure were prepared in UCSF Chimera.


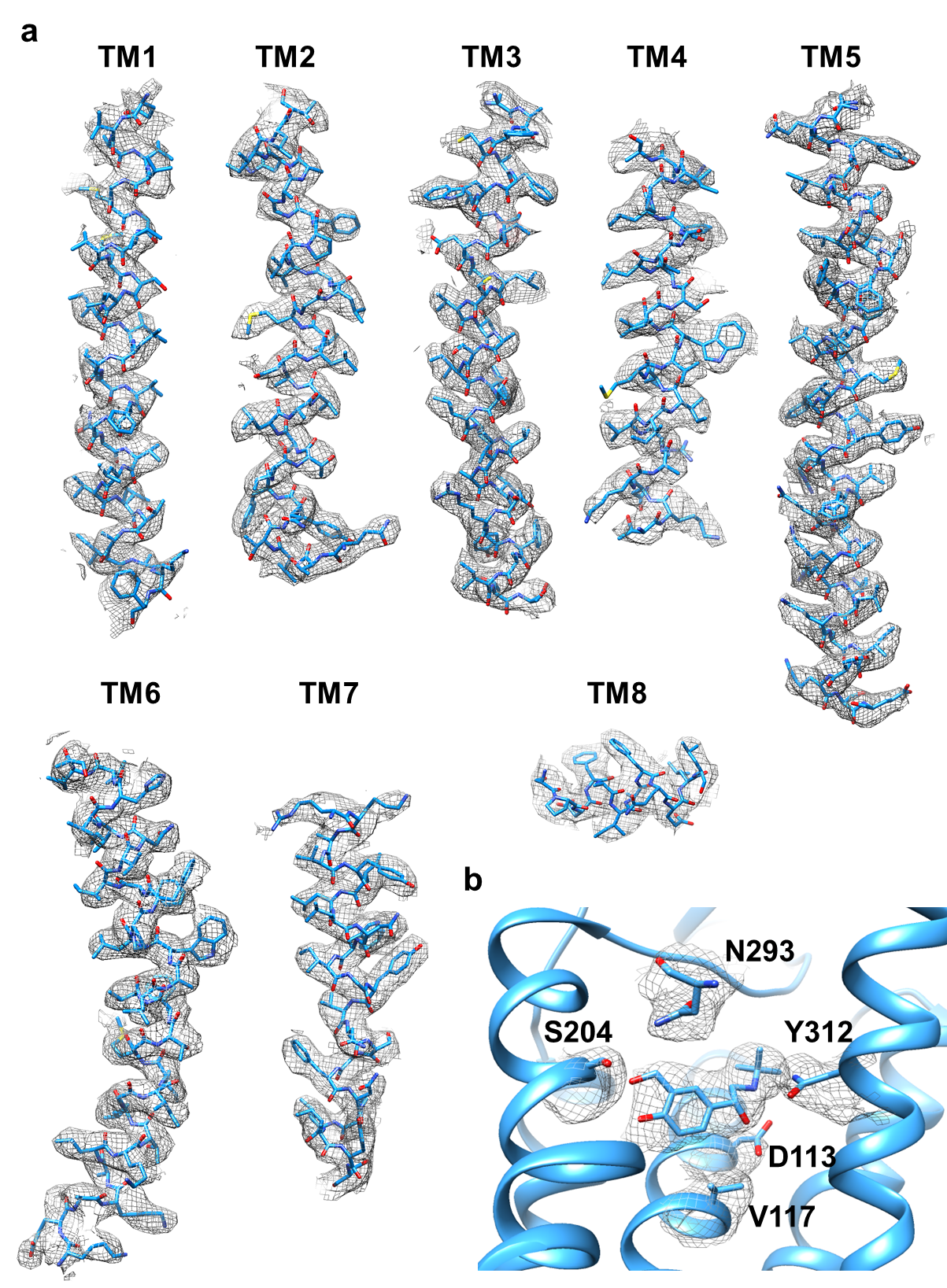


**Figure S4. Cryo-EM densities of representative segments of human salbutamol-β_2_AR-Gs complex.**

1. Cryo-EM density maps and models of representative segments of salbutamol-β_2_AR-Gs complex, TM1-TM8. The cryo-EM maps are shown in mesh and molecular models as stick representation.
2. Local density map of the salbutamol binding pocket.


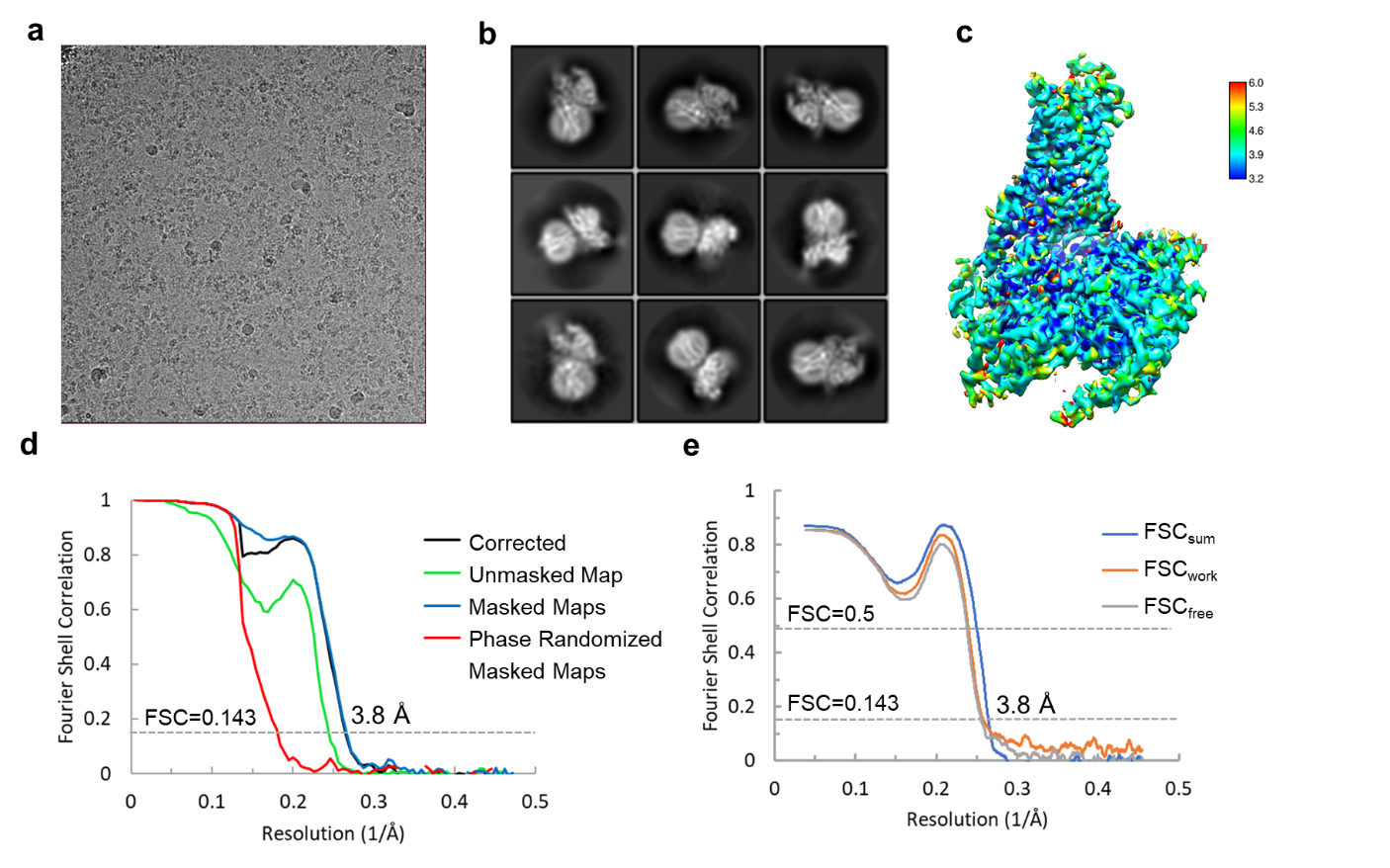


**Figure S5. Cryo-EM data analysis of human isoprenaline-β_2_AR-Gs complex.**

1. A representative cryo-EM micrograph of isoprenaline-β_2_AR-Gs complex after motion correction and dose weighting.
2. Representative 2D class averages of isoprenaline-β_2_AR-Gs complex showing distinct secondary structure features.
3. Cryo-EM density map colored by local resolution estimation using Resmap.
4. The gold-standard Fourier shell correlation curve of isoprenaline-β_2_AR-Gs complex indicating the resolution at the FSC=0.143 is 3.80Å.
5. FSC_work_/FSC_free_ validation curves of isoprenaline-β_2_AR-Gs complex.


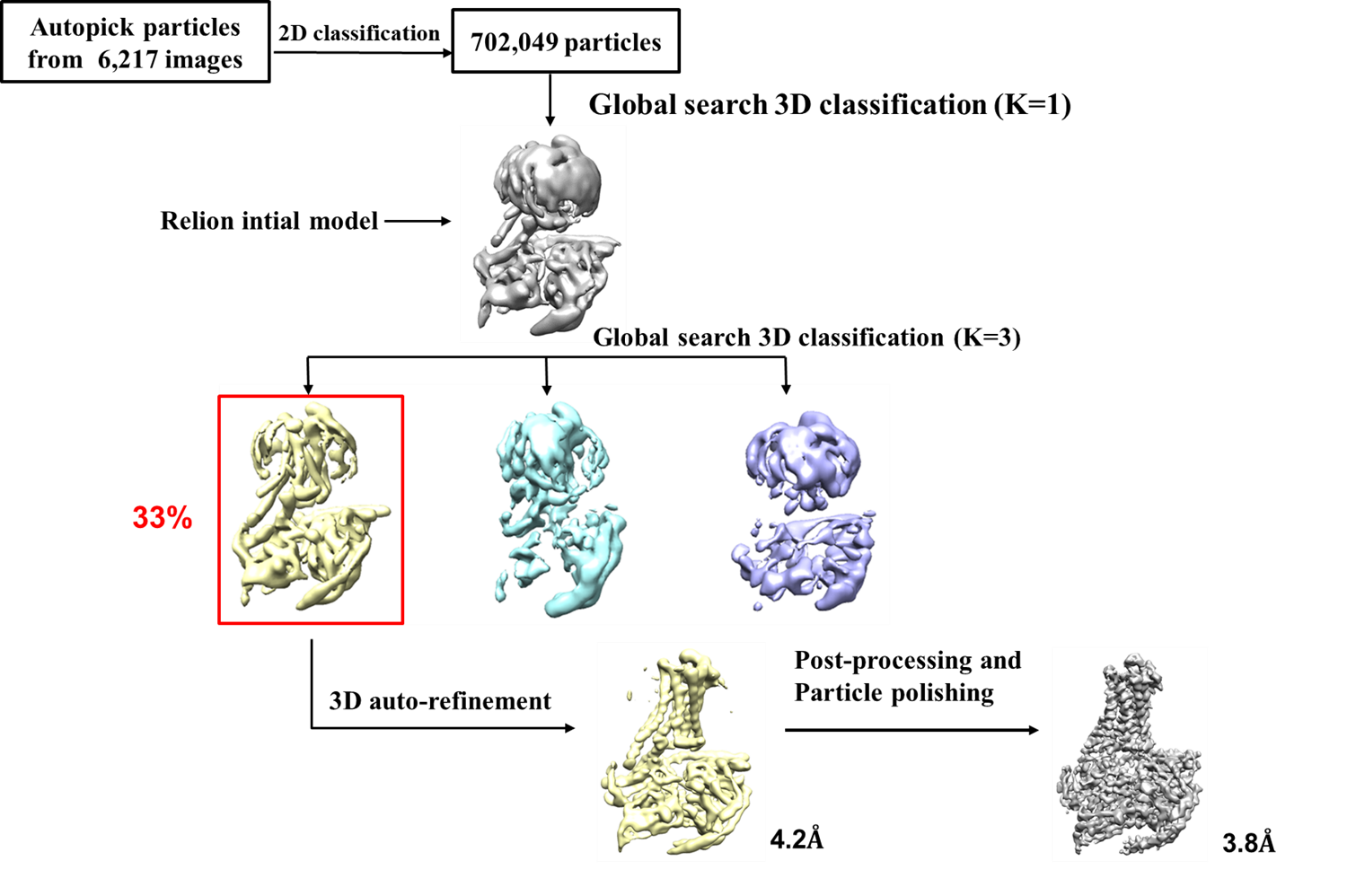


**Figure S6. A flowchart for the cryo-EM data processing and structure determination of the isoprenaline-β_2_AR-Gs complex, including particle projection selection, classification and refinement.**

The final reconstructed density map has an average resolution of 3.8 Å. All the images in this figure were prepared in UCSF Chimera.


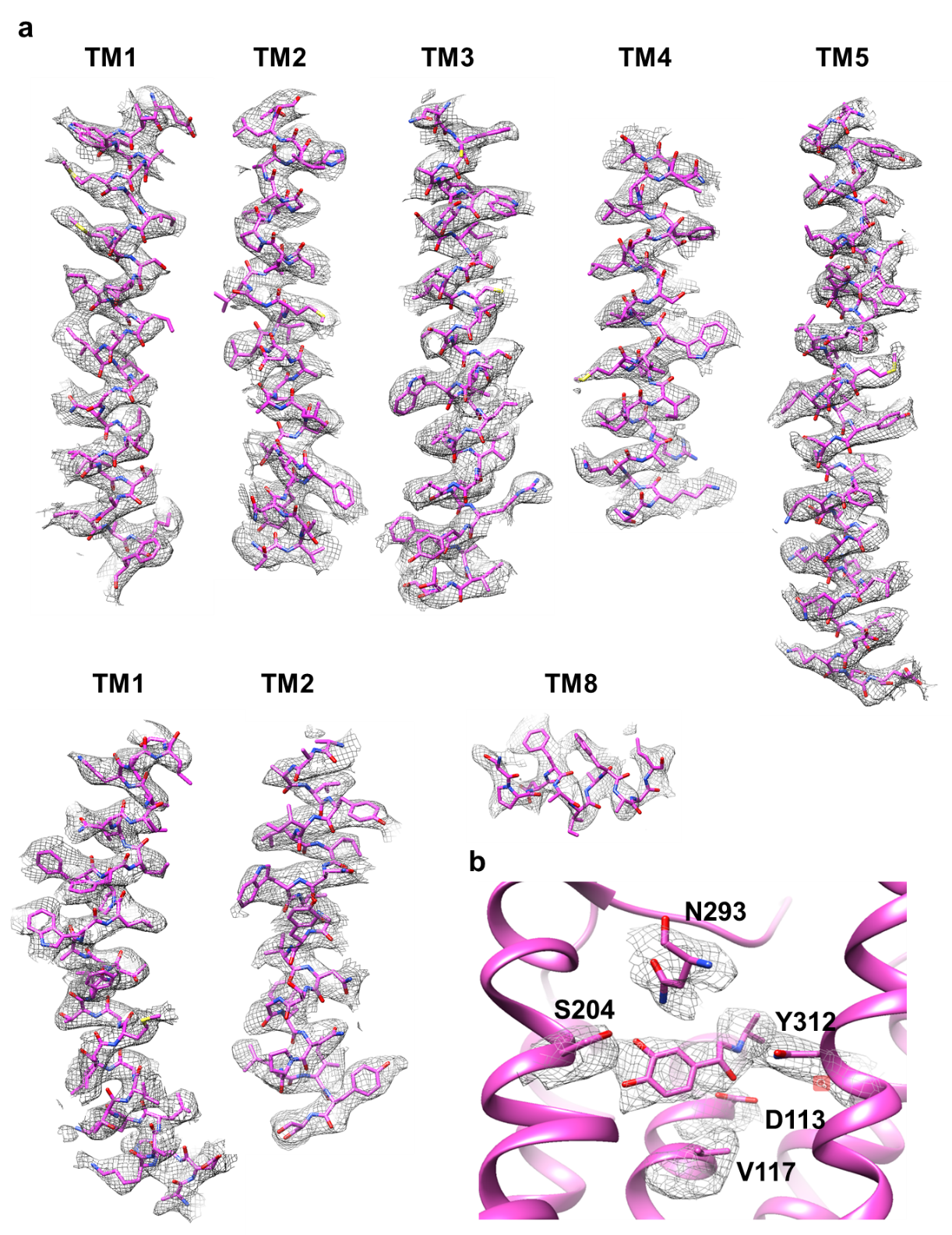


**Figure S7. Cryo-EM densities of representative segments of human isoprenaline- β_2_AR-Gs complex.**

**a** Cryo-EM density maps and models of representative segments of isoprenaline-β_2_AR-Gs complex, TM1-TM8. The cryo-EM maps are shown in mesh and molecular models as stick representation. **b** Local density map of the isoprenaline binding pocket of β_2_AR.


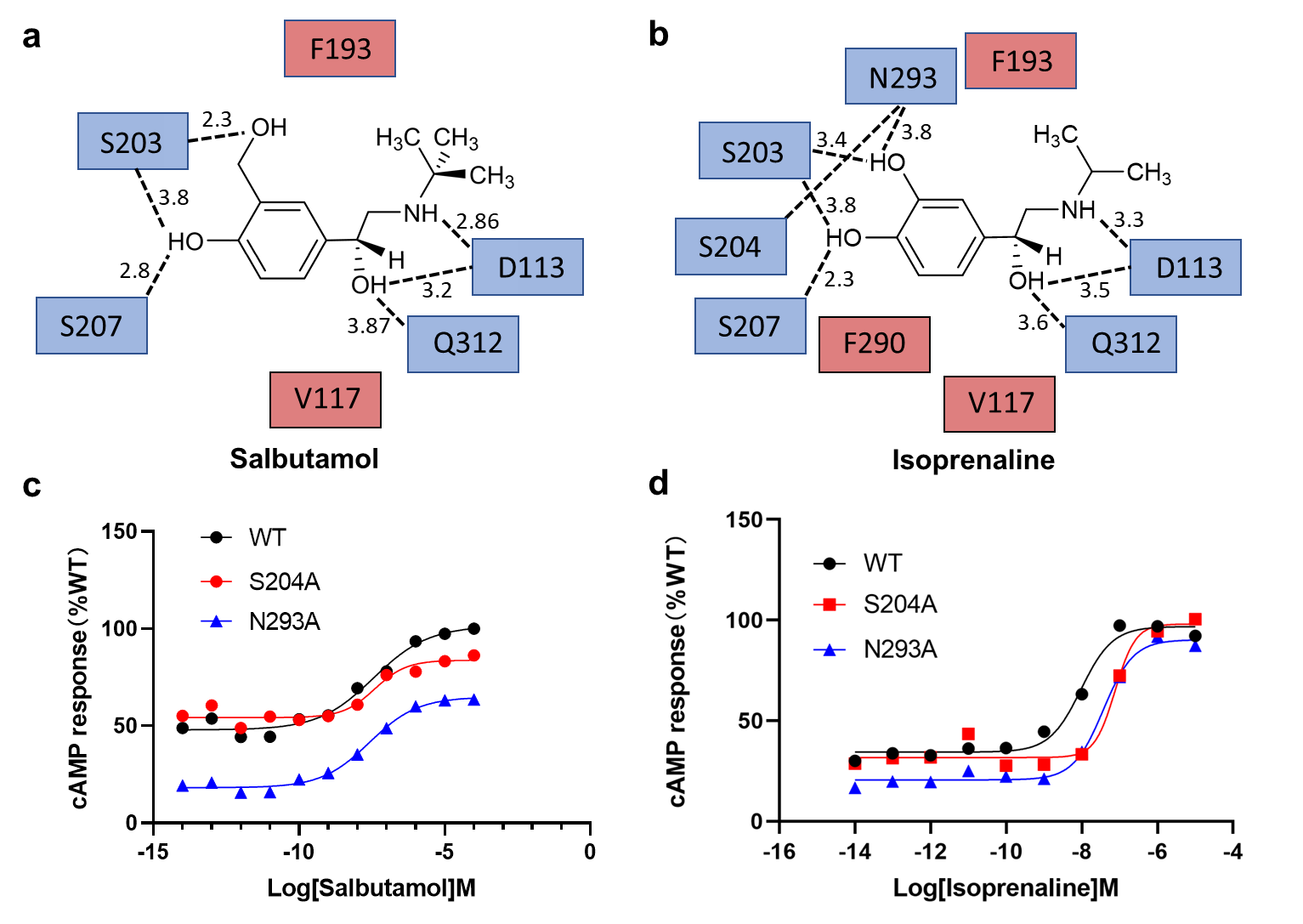


**Figure S8. Schematic representation of the interactions between the β_2_AR and the ligands.**

**a,b.** Display the amino acids that interact with the salbutamol**(a)** or isoprenaline**(b)**. Red boxes indicate potential hydrophobic interactions and blue boxes indicate potential polar interactions.

**c,d** cAMP accumulation analysis of wild-type β_2_AR and mutants. **(c)**The alanine substitution of residues N293 or S204 in binding pocket of salbutamol has little effect on the cAMP signalling assay. **(d)**Alanine mutagenesis of S204 and N293 in the isoprenaline binding pocket decease the activation of β_2_AR by isoprenaline.


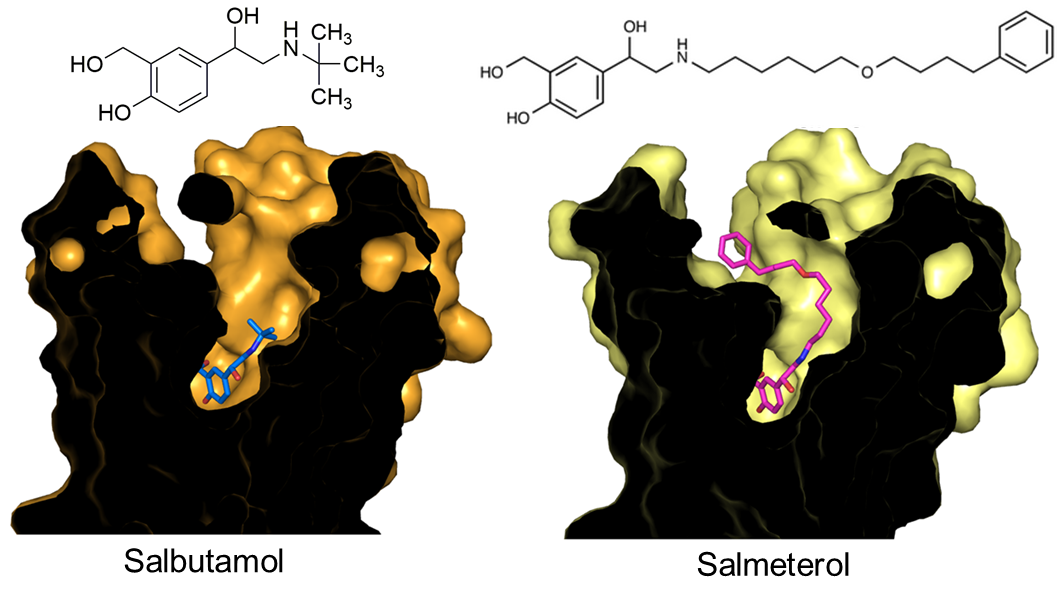


**Figure S9. Ligand binding interactions in the orthosteric site.**

1. Comparison of the ligand-binding sites between β_2_AR bound to salbutamol (blue sticks) and salmeterol (magenta sticks).
2. Sectional views of the orthosteric binding pockets of salbutamol (left) and salmeterol (right).


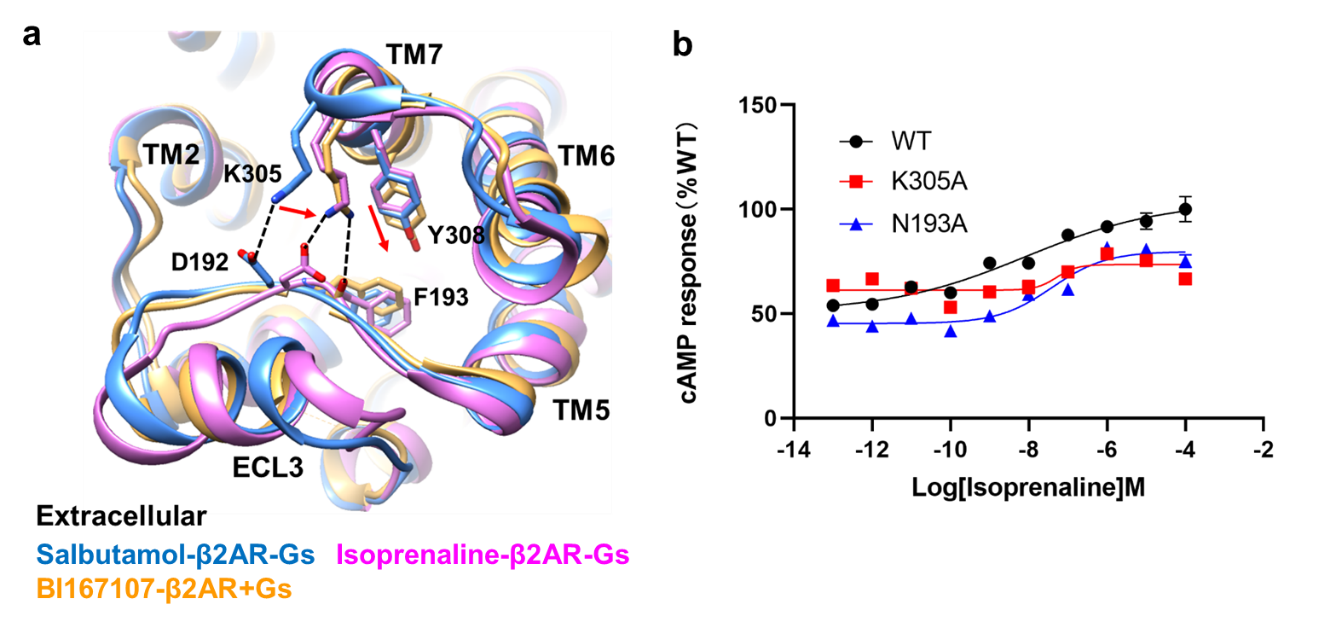


**Figure S10. Conformational changes on the extracellular side of the ligand binding pocket.**

1. Comparison of the extracellular views of the binding pocket between partial agonist salbutamol-bound β_2_AR (blue), full agonist isoprenaline-bound β_2_AR (magenta) and BI167107-bound β_2_AR receptor (orange).
2. Ala mutations of K305 or F193 in isoprenaline binding pocket induce the potency of β_2_AR in the cAMP accumulation assay.

**
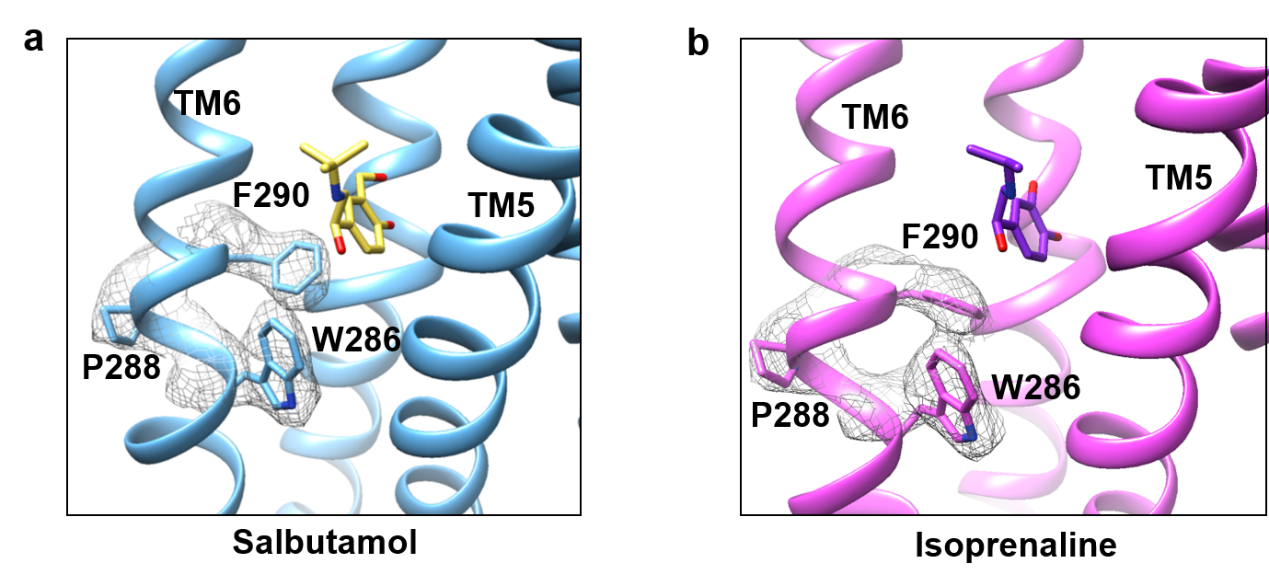
**

**Figure S11. The role of F290 in regulating the conformational changes of receptor TM6.**

**a,b** The local density map of the rotamer toggle switch in the salbutamol or isoprenaline-bound β_2_AR.


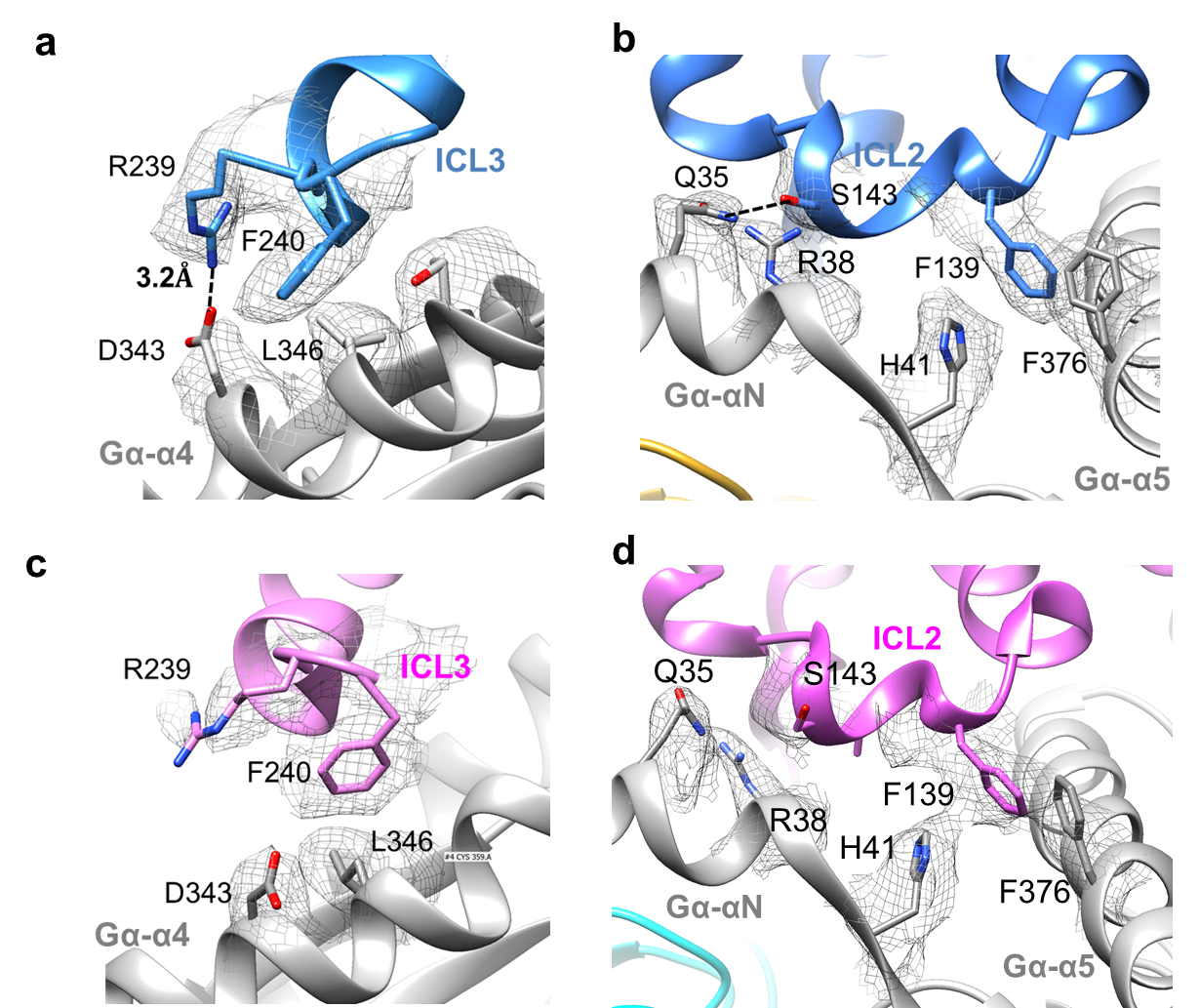


**Figure S12. The cryo-EM density map of residues located in the salbutamol bound β_2_AR-Gs interface and corresponding residues located in the isoprenaline bound β_2_AR-Gs interface.**

**
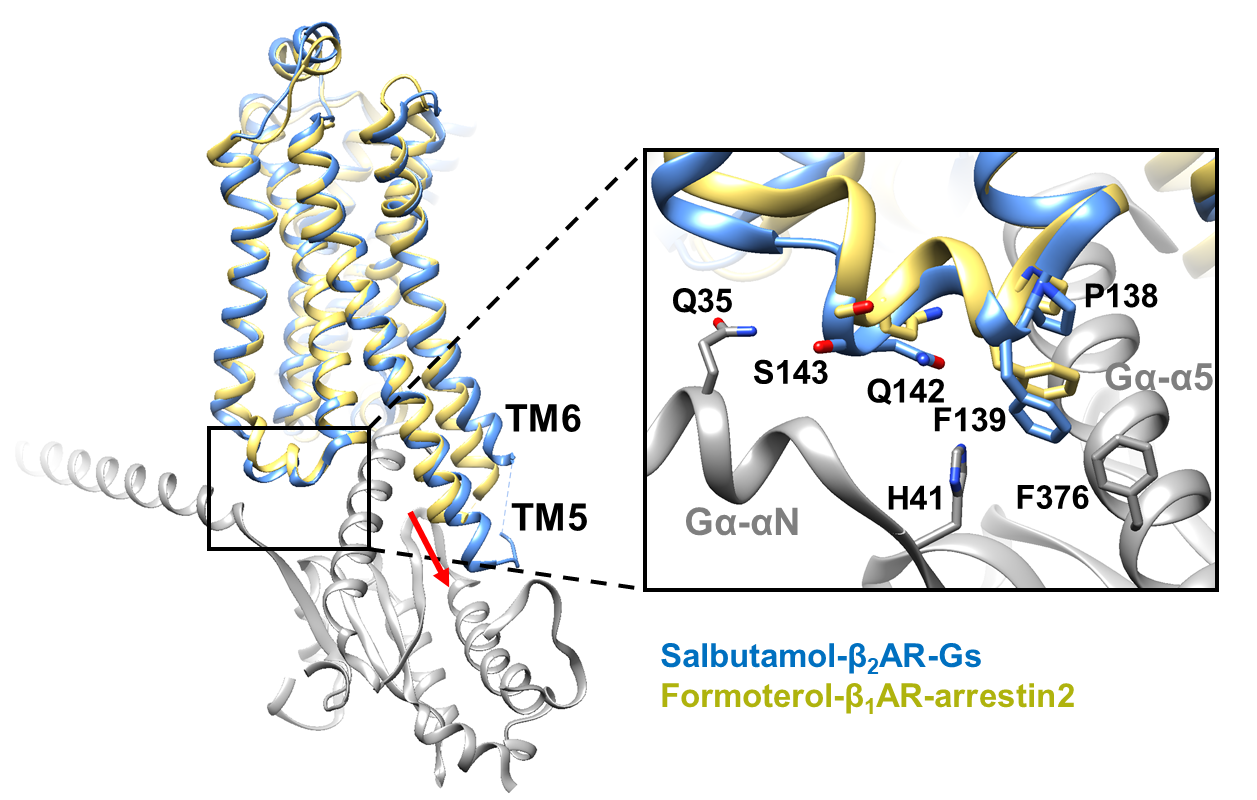
**

**Figure S13. Compared ICL2 conformation of salbutamol-β_2_AR-Gs complex with formoterol-β_1_AR-arrestin2 complex.**

The amino acids which can bind to both the Gs protein (F139, Q142, S143) and the arretin2 (S151, Q150, F147) are shown as sticks.

**Table S1.**

Statistics of cryo-EM data collection, 3D reconstruction and model refinement.

| **Data collection and processing** | |
| --- | --- |
| Protein | Salbutamol-β_2_AR-Gs complex |
| Microscope | FEI Titan Krios |
| Voltage (kV) | 300 |
| Detector | Gatan K2 Summit |
| Detector mode | Counting |
| Pixel size (Å) | 1.014 |
| Defocus range (μm) | -1.5~-2.5 |
| Electron dose (e^-^/Å^2^) | 57.6 |
| Magnification | 29000 |
| Number of images | 7026 |
| Symmetry imposed | C1 |
| Initial particle images(no.) | 921,057 |
| Final particle images (no.) | 455,803 |
| Map resolution (Å) | 3.26 |
| FSC threshold | 0.143 |
| Map resolution range(Å) | 2.056-999 |
| **Model refinement** |  |
| Model resolution (Å) | 3.26 |
| FSC threshold | 0.143 |
| Model composition |  |
| Chains | 6 |
| Ligands | 68H |
| Non-hydrogen atoms | 7,799 |
| Protein residues | 1,019 |
| R.m.s. deviations |  |
| Bond length (Å) | 0.004 |
| Bond angles (°) | 0.745 |
| Validation |  |
| MolProbity score | 1.60 |
| Clashscore | 4.37 |
| Ramachandran plot (%) |  |
| Outliers | 0.00 |
| Allowed | 5.61 |
| Favored | 94.36 |

**Table S2.**

Statistics of cryo-EM data collection, 3D reconstruction and model refinement.

| **Data collection and processing** | |
| --- | --- |
| Protein | Isoprenaline-β_2_AR-Gs complex |
| Microscope | FEI Titan Krios |
| Voltage (kV) | 300 |
| Detector | Gatan K2 Summit |
| Detector mode | Counting |
| Pixel size (Å) | 1.014 |
| Defocus range (μm) | -1.5~-2.5 |
| Electron dose (e^-^/Å^2^) | 57.6 |
| Magnification | 29000 |
| Number of images | 6217 |
| Symmetry imposed | C1 |
| Initial particle images(no.) | 702,049 |
| Final particle images (no.) | 231,827 |
| Map resolution (Å) | 3.80 |
| FSC threshold | 0.143 |
| Map resolution range(Å) | 2.056-999 |
| **Model refinement** |  |
| Model resolution (Å) | 3.80 |
| FSC threshold | 0.143 |
| Model composition |  |
| Chains | 6 |
| Ligands | 5FW |
| Non-hydrogen atoms | 7,337 |
| Protein residues | 986 |
| R.m.s. deviations |  |
| Bond length (Å) | 0.010 |
| Bond angles (°) | 1.201 |
| Validation |  |
| MolProbity score | 1.93 |
| Clashscore | 5.01 |
| Ramachandran plot (%) |  |
| Outliers | 0.00 |
| Allowed | 11.54 |
| Favored | 88.46 |

**Table S3.**

**Summary of EC_50_ values for the wild-type β_2_AR and β_2_AR mutants.** Curve-fitting was performed using the GraphPad prism. LogEC_50_ values represent the logarithm agonist concentration that produces half maximal response.

| β_2_AR | Salbutamol |
| --- | --- |
|  | Log EC50（M) |
| Wide Type | -7.59±0.05 |
| S204A | -7.57±0.04 |
| N293A | -7.53±0.06 |
| F290A | -7.23±0.15 |

| β_2_AR | Isoprenaline |
| --- | --- |
|  | Log EC50（M) |
| Wide Type | -8.03±0.09 |
| S204A | -7.02±0.03 |
| N293A | -7.79±0.81 |
| F290A | -6.67±0.38 |
| K305A | -7.36±0.65 |
| F193A | -7.42±0.45 |
